# Immigrant men ‘s perceptions and experiences of accompanying their partner for contraceptive counselling provided by midwives in Sweden- a qualitative study

**DOI:** 10.1371/journal.pone.0295796

**Published:** 2024-01-02

**Authors:** Mia Kolak, Anette Agardh, Christine Rubertsson, Stefan R. Hansson, Maria Ekstrand Ragnar

**Affiliations:** 1 Division of Social Medicine and Global Health, Department of Clinical Sciences, Lund University, Malmö, Sweden; 2 Department of Obstetrics and Gynecology, Skane University Hospital, Malmö, Lund, Sweden; 3 Department of Clinical Sciences Lund, Lund University, Lund, Sweden; 4 Department of Health Sciences, Faculty of Medicine, Lund University, Lund, Sweden; 5 Department of Clinical Sciences Lund, Lund University, Malmö, Sweden; 6 Department of Women’s and Children’s Health, Uppsala University, Uppsala, Sweden; Caleb University, NIGERIA

## Abstract

**Background:**

Male involvement in maternal health care has proven to be beneficial for improving maternal and child health and is often crucial in areas of family planning and contraceptive use. However, compared to male involvement in maternal health care, male involvement in contraceptive counselling is complex and controversial and thus faces certain challenges. Immigrant men in Sweden are often accompanying their partner for contraceptive counselling. Little is known about their presence and role.

**Aim:**

To explore how immigrant men from the Middle East and Afghanistan perceive and experience accompanying their partner for contraceptive counselling provided by midwives in Sweden.

**Methods:**

Inductive qualitative content analysis guided the interpretation of data based on 21 individual in-depth interviews.

**Findings:**

Balancing conflicting values and norms about sexual and reproductive health and rights including family planning was challenging and confusing when living in Sweden. Contraceptive counselling was perceived as a joint visit, and men were often acting as decision makers. The midwife’s role as a contraceptive counsellor was perceived as trusted, but knowledge was lacking about the Swedish midwifery model and the Swedish healthcare system. Providers’ ways of communicating sensitive information were crucial. Without marriage contraceptive counselling was unthinkable.

**Conclusion:**

Highlighting male engagement and including men’s sexual and reproductive health at policy levels are necessary for improving women’s sexual and reproductive health and rights. Additional and new ways of contraceptive counselling and midwifery services, such as outreach work and joint visits, are needed in order to reach both men and women.

## Introduction

Sexual and reproductive health and rights (SRHR) are described as fundamental to men’s and women’s health and survival, to economic development, to gender equality and to upholding human rights [1, 2]. Most often SRHR is considered to be a woman’s issue because of biological factors and socially defined gender roles that discriminate against women [1]. Each year there are about 121 million unintended pregnancies and about 79 million abortions, whereof at least 25 million are performed as illegal and unsafe abortions [3]. More than 350 million men and women need treatment for sexually transmitted infections and nearly 2 million people are newly infected with HIV each year [4]. Increased access to prevention including use of modern contraceptives and education for men and women, is needed in order to save lives and establish gender equality [4].

Being an immigrant is an important factor adversely affecting men and women’s sexual and reproductive health and rights, including contraception [5]. Immigrant status, lack of language skills, financial instability, and lower levels of health literacy are some of the barriers immigrants face in order to access health care including sexual and reproductive health care in the new home country [6]. Low trust in healthcare providers and low trust in general towards the society affect how the health care is perceived, which in turn affects people’s health [6].

Sexual and reproductive health care is described as even more trust sensitive and challenging than health care in general since it is closely associated with values and norms [7]. A recent study on values of non-western immigrants in Sweden found that their views about sex before marriage, contraception, abortions and other SRHR issues were in sharp contrast to those of the majority population in Sweden (17). Festinger [8] posited that holding beliefs, values, or attitudes that are diametrically opposed to behavior and to one another could give rise to internal psychological discomfort within people and thereby cause mental health related issues.

Sweden has a long history of migration [9]. In recent years the number of foreign-born immigrants has increased, now representing about 20 per cent of the total population [9]. The past 20 years of wars and conflicts around the globe have resulted in an increase of refugee and asylum-seeking migrants to Sweden, mainly from the Middle Eastern regions and Afghanistan to Sweden. The increase culminated during 2016 when 163 000 people mostly from Syria arrived in Sweden. During the same period a large number of unaccompanied children (mostly boys) and men from Afghanistan migrated to Sweden from conflict zones. Overall, during most of the 21st century, migrants to Sweden have predominantly been men [9].

For many immigrants the midwife is often the first contact with the Swedish health system. Previous research shows that this first healthcare experience is crucial for future visits and for trusting the healthcare system [10]. The midwifery profession in Sweden plays a vital role in the Swedish society, providing sexual and reproductive health care and promoting reproductive rights including advocating for gender equality [11, 12]. Midwifery is an important part of Sweden’s commitment to achieving the Sustainable Development Goals (SDG’s) set out in Agenda 2030 and for the right to receive universal health coverage [11]. The Swedish model with well-educated midwives providing sexual and reproductive health care throughout the woman’s entire reproductive life cycle is unique. The model has contributed to the lowest maternal and child mortality rates in the world [11]. However, the Swedish midwife’s professional role and responsibilities are not always well-known to the immigrant population in Sweden [13].

Lack of familiarity with the role of the midwives and the Swedish health system is a barrier for SRHR and for having adequate knowledge about where and how to access contraceptive counselling [14]. According to previous research, immigrant women in Sweden have less access to sexual and reproductive health care than native born women and have higher rates of unintended pregnancies, induced abortions and repeated induced abortions [15].

Since the beginning of the 1970’s, Sweden offers free access to contraceptive counselling and subsidized contraceptives [11]. Midwives working at maternal healthcare centers and at youth sexual health centers are the main contraceptive counselling providers and prescribers of contraceptives [11]. Despite this system, the unmet need for contraception in Sweden has increased from 9 per cent to 17 per cent in recent years [16]. Women’s unmet need has been defined by the World Health Organization (WHO) as the gap between women’s reproductive intentions and their contraceptive behavior [17].

Globally, male involvement in maternal health care has proven to be beneficial for improving maternal and child health [18] and is crucial in areas of family planning and contraceptive use [19]. However, compared to male involvement in maternal health care, male involvement in contraceptive counselling is complex and controversial and thus faces certain challenges [20]. Patriarchal structures, misconceptions, values and norms, and law and politics are some of the barriers that are affecting men and women’s access to and knowledge about contraceptives [1]. Previous research shows that non-western immigrant men in Sweden are accompanying their partners for contraceptive counselling and that their presence is significant and is affecting women’s contraceptive counselling and women’s use of contraceptives [13, 21, 22]. According to midwives providing contraceptive counselling and meeting immigrant women, men are accompanying their partners for contraceptive counselling, despite neither being the midwife’s patient nor her responsibility [21, 23]. Moreover, immigrant men in Sweden are more often acting as interpreters for their wives and to a greater extent serve as a primary contact with midwives than men in general in Sweden [24, 25]. There are few studies examining how immigrant men in Sweden perceive contraceptive counselling when accompanying their partner for contraceptive counselling provided by midwives [26]. To improve women’s sexual and reproductive health and in order to increase midwives’ understanding of the role of the accompanying partner, there is a need to understand immigrant men’s perspectives on accompanying their partners for contraceptive counselling provided by midwives in Sweden.

The overall aim of this study was to explore immigrant men’s perceptions and experiences of accompanying their partners for contraceptive counselling provided by midwives in Sweden. More specifically, we aimed to explore the following questions: What knowledge and perceptions do immigrant men have regarding contraceptives and contraceptive use? How do immigrant men perceive their own role in accompanying their partners for contraceptive counselling? Finally, what thoughts and opinions do immigrant men have regarding how contraceptive counselling can be optimized to better meet the needs of sexual and reproductive health and rights among immigrant women in Sweden?

## Method

### Study design

The study utilized a qualitative research design based on individual in-depth interviews. This approach was chosen to gain a deeper understanding and insight into Middle Eastern and Afghani immigrant men’s perceptions and experiences of contraceptive counselling and their role as accompanying partner for contraceptive counselling provided by midwives in Sweden.

### Study setting

The present study was conducted in Malmö, a city with about 350 000 inhabitants situated in southwestern Sweden. Malmö is the third largest and the fastest growing city in Sweden [27] with nearly half of the population under the age of 35 [28]. More than 184 nationalities are represented; where about 45 per cent of the population are of immigrant descent and about 30 per cent are foreign born [28]. Malmö is the city in Sweden with the largest influx of immigrants in recent years and with the largest proportion of non-European immigrants in relation to the population size [29]. Since the year 2015, the largest group of immigrants in Malmö originates from Syria followed by Iraq and Afghanistan [9]. Other countries of non-European origin commonly represented are Iran, Somalia, Palestine, and Lebanon [27].

### Data collection

Data collection took place from September through November 2021, with individual in-depth interviews conducted by the first author. A purposive sampling strategy was used [30] targeting immigrant men > 18 years from the Middle East and Afghanistan who had experience of accompanying their partner for contraceptive counselling provided by a midwife in Sweden. Furthermore, the men needed to be residing in Sweden, speak Swedish or English and, if needed be willing to use an interpreter.

Participants were recruited through i) two maternal health clinics in immigrant dense areas (one privately managed and one under the direction of the regional health authorities and ii) through the County Administrative Board’s regional civic and health communicator organization in Malmö.

Before recruitment, senior managers for the three settings were contacted by the first author who provided them with information about the study. After approval of the study from the senior management, midwives and interpreters working at the maternal health clinics and a head regional civic health communicator operated as gatekeepers facilitating contact between the first author and potential participants meeting the inclusion critera. The gatekeepers at the maternal health clinics provided oral information face-to -face, orally, to men who were visiting the clinics either together with their partner or alone in behalf of their partner. The gatekeeper from the regional civic health communicator organization provided information at a staff meeting in which the first author participated and informed about the study as well. Furthermore, information about the study was provided verbally by the civic health communicators’ gatekeeper in two classes at a public school for newly arrived immigrants, which was the workplace of the regional health communicators organization. After establishing contact with presumptive participants, the gatekeepers did not receive further information about who participated in the study.

During the recruitment process, the first author was situated on site at the different locations with permission from the respective senior management, alternating between the two different maternal health clinics and the public school for newly arrived immigrants, where the regional civic and health communicators were situated. This made it possible for all potential participants both at the maternal health clinics and the public school for newly arrived immigrants who had questions about the study or who had expressed their interest to participate in the study to establish a direct and personal contact and opportunity to, if time permitted, schedule or perform the interview directly on site. All together 21 interviews were conducted and included in the study (14 from the private clinic, 3 from the regional center and 4 that were recruited through the civic and health communicators).

Initially, two pilot interviews were conducted to test and evaluate the purposive recruitment and the interview guide. As no adjustments were needed and the pilot interviews were deemed to be of sufficient quality, they were included in the analysis.

All interviews were conducted until no new information emerged. The interviews were conducted at the maternal healthcare clinics in a secluded room with no one else present but the interviewer, the participant and the interpreter (where applicable) or at a secluded area of the participants’ own choice. One interview was conducted digitally via Zoom [31]. Fifteen interviews were conducted in Swedish, two in English and four interviews with the help of an Arabic interpreter (whereby three were with an ‘on site’ interpreter and one with the assistance of an interpreter by telephone).

The interviews started with general questions about the participants’ background, followed by open-ended questions concerning the individual perceptions and experiences of contraceptive counselling provided by midwives when accompanying one’s partner.

A semi-structured interview guide according to Kvale (27) was used covering the following areas: perception of one’s role as accompanying partner, communication and language, relation to and expectations concerning the midwife, pre-understanding and knowledge about contraceptives and contraceptive counselling, and perceptions of accessibility and needs in relation to the midwife and the contraceptive counselling. Open-ended questions were used, such as “What expectations did you have when accompanying your partner for contraceptive counselling provided by a midwife?” or “How did you experience the information that was given?”. Follow-up questions were asked, such as “Can you tell me more about your own role?” or “Can you describe how you experienced the relation with the midwife?”. The length of the interviews varied between 25–75 minutes (median 40 minutes). All interviews were digitally recorded and transcribed verbatim and then translated into English by the first author.

### Ethical considerations

Both gatekeepers and the interviewer informed participants about the purpose of the study. Prior to the interview, written and oral consent was obtained, and participants were informed that participation was voluntary, that they could withdraw at any time, that only the researchers would have access to the data and that all collected data would be presented with no risk of any individual being identified. To ensure further anonymity, each study participant was assigned a code number (P1 –P21). The study was approved by the Regional Research Ethics Board, Sweden (Dnr: 2021–04100).

### Data analysis

**Fig 1:**
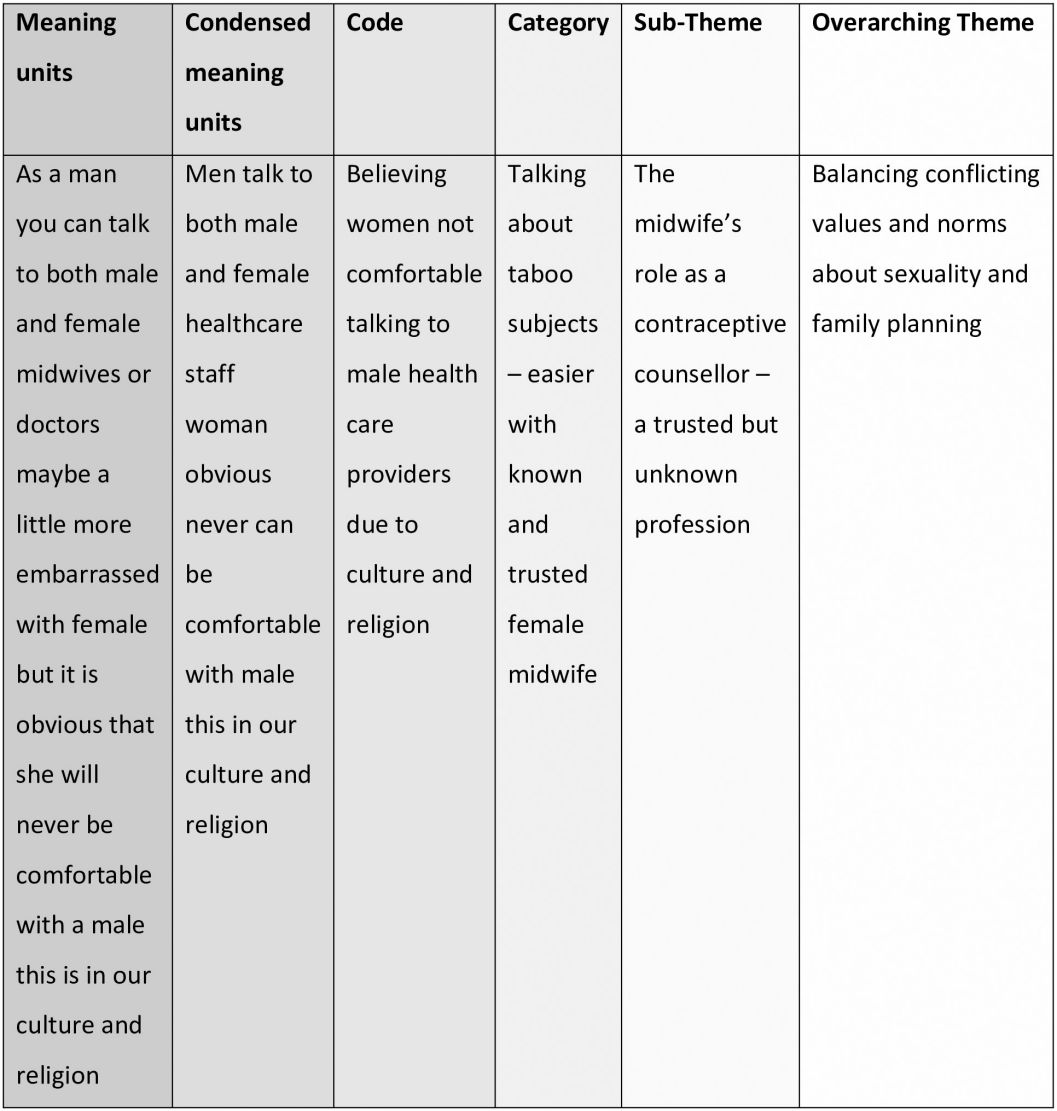
Example of the analytical process.

The data was inductively analyzed by manifest and latent qualitative content analysis as described by Graneheim and Lundman [32]. NVivio (version 1.6.1) software program was used for organizing data throughout the analytical process [33]. The transcribed interviews were read through by the first, second, and last author several times to gain a sense of the whole and in order to understand the content of the data in relation to the overall aim. Next, the first author identified relevant meaning units. The identified meaning units were shared among the first, second, and last author in order to strengthen the credibility of the analysis and in order to create a shared view of the process and the data content. This was followed by condensation of the meaning units and coding which was done by the first author. The codes were read through several times and verified by the first, second, and the last author to avoid lone research bias. The first and last author then discussed and sorted the codes jointly into content areas based on the study aim and the thematic study guide in order to gain consensus. Codes with a similar content were grouped together into manifest categories. The first and last author moved back and forth between the interview transcript and the coding to ensure that the coding process was satisfactory. Discussions among the first, second, and last author were held throughout the entire analytical process to obtain a thorough level of shared understanding of the interpretation of the findings. The transcripts were then read through again for verification. Furthermore, throughout the ongoing process of continuously analyzing and reading through the codes and categories, the latent sub-themes and overarching theme emerged. Fig 1 shows an example of the analytical process.

## Results

### Demographic background of the participants

The demographic background of the participants is presented below. The participants were characterized by diversity in socio-economic background and length of residency in Sweden (See Table 1).

**Table 1:** Study participants (N = 21).

Gender:	Male
Age:	27–62 years.
	Median: 37 years.
Countries of origin:	Afghanistan, Syria, Lebanon, Iraq, Jordan, Palestine, and Quwait.
Years of residency in Sweden:	1–30 years
	Median: 7 years
Education:	No schooling n = 1
	Primary education, n = 10
	Higher than primary education, n = 10
Working experience:	Unemployed, n = 8
	Studying, n = 5
	Employed, n = 8
Civil status:	Married, n = 20
	Divorced, n = 1
Partner’s parity:	Children, n = 19 (> 2)
	No children, n = 2
Religions mentioned:	Islam

**Fig 2:**
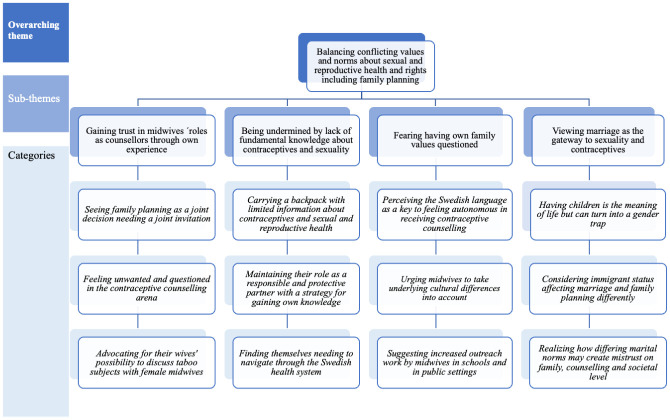
Overview of the results.

The analytical process resulted in one overarching theme, four sub-themes, and 12 categories. An overview of the results is presented in Fig 2 below and described further in the text.

### Balancing conflicting values and norms about sexual and reproductive health and rights including family planning

The overarching theme reflects how the participants were trying to find their own way regarding family planning and use of contraceptives in relation to Swedish law and Swedish values and norms about sexual and reproductive health and rights. Coming to Sweden, a country with open attitudes and, as described by the participants, a free and democratic way of viewing sexual and reproductive health and rights and contraceptives was described as difficult. It created confusion and insecurity within families and among individuals. The participants felt challenged by the midwives and the Swedish healthcare perspective, as they represented another sexual discourse than their own. Adaptation to the Swedish society was often difficult and made the participants feel as if they were balancing between two different cultures.

#### Gaining trust in midwives’roles as counsellors through own experience

This sub-theme describes the participants’ lack of knowledge about the Swedish midwife’s professional role and abilities. Knowledge about the midwife’s role in Sweden was usually shared among friends and family who had previous experiences or acquired through contact during pregnancy. When knowledge about the Swedish midwife’s professional role was acquired, the midwife was perceived as the overall trusted and preferred source of information. Furthermore, a midwife was described as a female caregiver, never a male. Moreover, according to the participants, information about contraceptives and women’s sexual and reproductive rights was considered more reliable if it came directly from the midwife rather than if the wife referred to what she and the midwife had been discussing during the consultation. Since use of contraceptives was not solely the wife’s individual decision, the men felt a need to be directly involved in the counselling in order to be a part of the decision.

*Seeing family planning as a joint decision needing a joint invitation*. Participants felt that if and when to have children was a joint decision between husband and wife, and they questioned why husbands were not automatically included in the invitation for contraceptive counselling by the midwife in Sweden. The purpose of contraceptive counselling and use of contraceptives was described as a mutual way to plan their future together with their wife. Furthermore, the participants stated that in most relationships it was not uncommon that the husband is the one who decides about the use or non-use of contraceptives, sometimes resulting in women using contraceptives without their husband’s knowledge. Some of the participants described that not being invited as a couple created suspicion towards the Swedish health system and the midwife and raised questions about what kind of information was to be given. Joint visit invitations would have created a feeling of security, showing that the husband was included. It would have reduced the feeling of being set aside and worries about the wife receiving inappropriate information which was not in accordance with the couple’s values and norms. Participants referred to equality between genders according to Swedish gender politics and argued that a joint invitation could have the potential of changing norms and creating opportunities for equal growth between women and men. Furthermore, the participants thought it would increase men’s understanding about women’s sexual and reproductive health and rights if they came for a joint contraceptive counselling session, which in turn would increase women’s access to the same rights.

“*The invitation must be for both…*.*not just for the woman alone…*.*then you offer a visit as a family…*.*I think that is a good beginning…a good way…to talk about this…*.*it also creates security*, *and the man does not feel in any way excluded or threatened or that there is something going on with the woman from the “underneath” … you know getting inappropriate information…*”

(P10)

*Feeling unwanted and questioned in the contraceptive counselling arena*. The participants perceived an underlying demand from the midwives and the Swedish health system that it was mandatory for them as a husband to be present when their wife was giving birth. However, during contraceptive counselling this was neither expected nor accepted. The participants described how they had been questioned by midwives when accompanying wife for contraceptive counselling and that the visit was not as friendly and informative as expected. Some felt that the midwife was protective of the wife, not understanding the reason why the husband was accompanying. Being an immigrant and having a lack of knowledge about contraceptives and experiencing language barriers created a feeling of insecurity in the meeting with the midwife, not knowing what the expectations of them were from the midwife. Some of the participants stated that midwives and other healthcare providers generally viewed men from the Middle East as being oppressive and judgemental towards women. The contraceptive counselling visit was perceived as foremost about women’s rights and autonomy, and the midwife neither needed nor requested support from the accompanying husband. The participants described that they had often been the first man in their family who had accompanied their wife when giving birth and that it was a very positive experience. For them, there was no difference between accompanying a wife for giving birth or for contraceptive counselling; it was their family life.

*“…I was chased away from the midwife clinic even though I knew the midwife and my wife knew her too…*.*but she (the midwife) yelled at me… “why am I going with her (the wife)*?*”*….*but why does she say that*?*… At the same time at the birth unit…*.*"where are you*? *come here…take care of your child…*.*you should be here…why are you not here*?*"… you know…it’s…in Sweden you have to be present at births*…”

(P5)

*Advocating for their wives’ possibility to discuss taboo subjects with female midwives*. The importance of meeting a trusted female midwife providing contraceptive counselling was emphasized. Use of contraceptives and sexual and reproductive health and rights in general were described by the participants as taboo subjects and something they did not talk about openly and freely. Newly married couples would sometimes not talk about contraceptives even with each other, and therefore unplanned pregnancies occurred. The participants believed that their wife would find it easier and more comfortable to discuss contraceptive use with a female midwife rather than with a male healthcare provider, including doctors. They felt challenged by having to navigate in the Swedish healthcare system, for example by not being able to make the wife see female healthcare providers only. On the other hand, the participants stated that if men were to receive more factual information about anatomy and reproduction they believed attitudes and understanding would potentially change, which would in turn increase women’s access to sexual and reproductive rights, including seeing both female and male healthcare providers. Others explained that male staff was not wished for, but had to be accepted, and religion and tradition needed to be set aside in Sweden. However, the participants stated that after having received contraceptive counselling by a familiar and trusted midwife in Sweden, their general knowledge about reproductive health and sexual and reproductive health and rights was increased, and they started to discuss and talk about these matters with their wives.

… “*It was a lot of stress on me who is a man…coming from this background… religion and culture… the staff…they haven’t talked about this with me at all before…that there was a risk of meeting a male caregiver…but it was necessary for her sake*, *so myself I said*, *"it’s okay”*, *but it did not feel ok… and I know for others… it’s not okay at all*” …

(P9)

#### Being undermined by lack of fundamental knowledge about contraceptives and sexual and reproductive health and rights

This sub-theme captures the participants’ perceptions of having a generally poor level of knowledge about contraceptives and sexual and reproductive health and rights and their need to know more. Some of the participants described that it was after arriving in Sweden that they had heard about contraceptives for the first time through reading tabloids or by searching the internet. The length of time in Sweden and knowledge of the Swedish language were crucial aspects influencing the knowledge level about contraceptives.

*Carrying a backpack with limited information about contraceptives and sexual and reproductive health*. According to the participants, they had received no or little school-based education regarding reproduction in their home countries. Sexual and reproductive health and rights had not been taught at all. Some of the participants had never heard of contraceptives when growing up and they had never heard people talking about contraceptives openly. Knowledge was shared between friends and discussed with other men. Knowledge about contraceptives was described as limited to general knowledge regarding some specific methods such as intrauterine devices, implants, contraceptive pills, and condoms. The participants also revealed they had scarce knowledge regarding reproduction, fertility, purpose of contraceptives, working mechanisms of contraceptives, and their side effects. Men’s fertility was perceived as being less questioned in general and that this partly explained why the use of and choice of contraceptive methods were seen as the woman’s issue and responsibility. Condoms were mainly described as a trusted method for protecting against pregnancy and were generally not associated with protection against sexually transmitted infections (STIs). The knowledge level about STIs and HIV was described as even lower than their knowledge about contraceptives.

*…” Before I came to Sweden*, *I had little knowledge… even if I am a highly educated person our culture and religion does not allow this knowledge…I knew little about condoms only … that this was for protection from pregnancy…but not for protection for some diseases… I had never heard about sexual diseases… or how you really can get HIV” …*

(P19)

*Maintaining their role as a responsible and protective partner with a strategy for gaining own knowledge*. The participants stated that they felt responsible for their wife’s wellbeing and that their role was to accompany their beloved wife as a supporter and a guardian, protecting her from feeling alone and vulnerable in a meeting with an unfamiliar midwife talking about a sensitive and private subject. Participants who were married to women who had grown up in Sweden and/or to women having more knowledge than themselves stated that it made them feel insecure and shy not knowing what was expected of them at the contraceptive counselling visits and in relation to the midwife. Those who were married to women having no or little knowledge felt that it was their responsibility to gain knowledge in order to help them as a couple. According to the participants, their limited knowledge about contraceptives could lead to misconceptions that contraceptives could harm their wife physically or that it was against their religious beliefs, which in turn could lead men not to allow their wives to use any contraceptives. The participants wanted it to be mandatory for them to accompany the wife for contraceptive counselling for at least one or two visits and/or to have own access to a midwife to increase their own knowledge levels.

*…” I always accompany my wife for everything…it is only the two of us here…we like going together…feeling more secure…we were not sure what kind of information was going to be given…but mostly we both needed knowledge…especially when we were new here… our midwife taught us so much about contraceptives*, *about women*’*s health and…about Sweden” …*

(P8)

*Finding themselves needing to navigate through the Swedish health system*. According to the participants, it was difficult to find out where to acquire contraceptive counselling when being new in Sweden. The Swedish healthcare system, including maternal health clinics, was described as being difficult to navigate through and as being difficult to access without having previous experiences of how the system was working. Some of the participants described how they had called the ambulance for getting help for their wife, not having knowledge about where to get adequate health care. The participants felt that the Swedish health system was slow and time consuming and that it was not possible to approach a midwife or other healthcare staff for asking quick questions without going through formal procedures. The participants described that it was shameful to ask for help regarding intimate regions especially for women. Therefore, to avoid unintended pregnancies and health issues it was even more important that sexual health literacy was improved for both men and women and that they should not get lost in waiting lines. When participants did not get quick help from midwives or health care, they turned to health clinics that had staff with the same ethnic and cultural background as themselves, finding such clinics to have more flexible opening hours. Furthermore, the participants described that these local health clinics understood the importance of easy access to health care and the way that such care should be provided, including cultural and religious understanding about contraceptive use.

…” *the local ethnic clinics…they understand when you ask for quick answers …it is not better quality…but it is sometimes easier… because we also share the same feelings about how to handle these sensitive things…talking about women*’*s issues”* …

P14

#### Fearing having own family values questioned

This sub-theme depicts the importance of communication and use of language when talking about contraceptives and sexual and reproductive health matters. The participants described that it was important *how*, in what words, the communication about contraception and sexual and reproductive health and rights was provided in order to receive the information positively. The participants stressed the need for established relational trust with the midwives and the importance of feeling secure when communicating during the contraceptive counselling. Appropriate communication was described as a way for the midwife to show the husband and wife that there was no intention of destroying family values.

*Perceiving the Swedish language as a key to feeling autonomous in receiving contraceptive counselling*. The Swedish language was perceived as the most crucial factor for being able to find information and for being able to receive contraceptive counselling. Looking for information on the internet and searching for information were difficult, not knowing the Swedish language. The participants felt that it was important for them not to use interpreters if they themselves could translate. Contraceptive counselling was described as a matter between husband and wife, a private subject; therefore, there was no reason for using an outside interpreter if the language level was sufficient. Some of the participants were satisfied and had good experiences of using interpreters initially when arriving in Sweden. However, as soon as they could speak a little Swedish, they preferred to manage on their own. It was not only a matter of feeling independent. There was also a general mistrust described towards interpreters, both regarding confidentiality and accuracy in translation, which some of the participants had personal experiences of.

…” I believe men in general are feeling embarrassed talking through an interpreter…it is not an optimal situation…there is always a possibility the interpreter knows the family…spreading rumors…and …the interpreter is disrupting the focus from the topic and from the situation…it is always better to manage without when you can” …

P5

*Urging midwives to take underlying cultural differences into account*. Midwives were described as a trusted profession that should be able to communicate and provide knowledge to persons of all ages. Furthermore, it was stated that there was a need for an underlying understanding among midwives of religion and culture and that it was important for midwives to know how to present and provide information to avoid and to reduce stigmatizing barriers. According to the participants, religion and culture permeates everything and cannot be separated from the individual. Nevertheless, this does not mean that people cannot receive information about contraception and sexual and reproductive health and rights. However, it needs to be done in a tactful and sensitive manner. For example, information about sexual and reproductive health and rights and/or contraception was sometimes found to be delivered too directly and thus in an ‘inappropriate’ way. The participants described how they felt chocked when realizing that in Sweden, general information about contraceptives and sexual and reproductive health and rights was given to adolescents and sometimes even to children from early ages. Participants felt that it was necessary for the midwives and other SRHR communicators in the Swedish society to adjust the ways of communication and at what age to initiate talking about sensitive and taboo topics. The importance of how, in what words and ways, information was to be given in order not to frighten or offend the recipient was stressed.

…”*and this thing how language is used is really important…you often hear doctors and midwives…spontaneous sentences…simple sentences and it doesn’t feel good…this is about my wife…you know all these terms*, *you can say them in another way…not so directly…you know show on paper and say that this and this so directly about her life…No*, *you have to say it nicely…had it been about myself then ok but now it’s about my wife who is my everything…she’s all I have…you know it’s like in our culture…we don’t talk directly to a woman about things like that…but here you always talk like that about everything*…"

P15

*Suggesting increased outreach work by midwives in schools and in public settings*. Participants described that outreach work was conducted in their home countries to spread information about health and they questioned why they have not seen similar public health campaigns in Sweden regarding midwives’ role in Sweden, contraceptives, and sexual and reproductive health and rights. Furthermore, midwives were the preferred providers of information about sexual and reproductive health and rights for newly arrived immigrants and in Swedish schools, rather than having such information provided by civic health communicators and teachers at language schools. The participants described that knowledge about the midwife’s role, contraceptives, and where to access contraceptive counselling should additionally be provided at public places such as at the Swedish Public Employment Service, the Swedish Tax Agency, and at Primary Health Centers. Facebook groups and other social media were described as sources where useful information could be provided and many people could be reached.

…” *you have to spread the knowledge and create security about this topic… you do that by reaching out to people…sometimes in our countries they do public health campaigns…*.*here you could do campaigns on Facebook… we have for example big groups for people from Syria that could be reached…or other platforms that people now use a lot”*…

(P7)

#### Viewing marriage as the gateway to sexual and reproductive health and rights and contraceptives

This sub-theme illustrates how marriage was seen as an institution and a central part of life. Only through marriage could sexual relations be accepted, and marriage led to a need for contraceptives. As sex before marriage was described as forbidden and unaccepted by cultural, traditional, and religious beliefs and also strictly regulated by law in the participants’ home countries, contraceptive counselling was generally not legally accessible before marriage. Marriage was described by the participants as a form of control and protection against promiscuity for both men and women. Living in Sweden with liberal sexual and reproductive health and rights values and norms created a feeling of confusion and a sense of "losing the inner moral compass”. Information was described as ideally to be provided when living in the actual and correct context for use of contraceptives.

*Having children is the meaning of life but can turn into a gender trap*. Children were seen as an important, central, and natural part of life and in connection with marriage. This was described as a strong and traditional value, but having large families with many children was perceived as being more challenging in Sweden compared with the participants’ countries of birth. Abortion, however, was not viewed as an option due to religious beliefs. Unplanned and/or unintended pregnancies were, according to the participants, affecting the family relations and economy. Traditionally, most often the husband’s role was that of the family’s sole breadwinner, but such a role turned out to be difficult in the new home country. The participants described that they were surprised that the Swedish law and social welfare system did not encourage women to use contraceptives. Instead, the Swedish welfare system with a generous child support was viewed as a system encouraging women’s bodies to be used for reproductive matters, enabling families to receive financial support and high living standards without the woman ever getting into the labor market, learning the Swedish language, or using any contraceptives for family planning. This welfare system was described by the participants as not promoting gender equality, which they believed was a strong Swedish value.

…” *sometimes it is a way of living…not to use contraceptives…maybe it is religious…but I know…it is difficult to learn a new language…difficult to get a job…and the Swedish welfare system is generous…so it is easy to have more children…but this is creating baby machines and this is not gender equality*” …

P11

*Considering immigrant status affecting marriage and family planning differently*. The participants stated that their immigrant status and re-settlement in Sweden had a significant impact on how they planned for their family. Detached from family and friends from their countries of birth, the participants felt a strong bond between husband and wife, being just the two of them without the rest of the families in Sweden. This affected their view of family planning. Some of the participants stated that it was important to have a legal right to stay in Sweden before having too many children and therefore they as a couple needed information and access to contraceptives upon arrival. Others meant that due to their legal status it was important not to use contraceptives to create a family in Sweden and thereby hopefully increase their chances to stay in Sweden. Some of the participants stated that they had been migrants in several countries and that they experienced that there was a general lack of information from host countries to migrants about how and where to access contraceptives and contraceptive counselling. However, the participants stated that there was a difference in views about family planning and use of contraceptives if one member of the couple, the husband, or the wife, had settled in Sweden first or had been born in Sweden. This created a feeling of security and enabled family planning from a personal perspective unrelated to immigrant status.

*"…you know…my wife she wanted to use contraception*, *but we didn’t know if we could stay in Sweden or not…but I told her that even if we stay or not*, *baby is always welcome…God decides how many children we are having…but I know she used sometimes and I found pills and threw them away” …*

P20

*Realizing how differing marital norms may create mistrust on family*, *counselling and societal level*. Coming from collective clan structures with strong marital norms affected attitudes towards access to contraceptives and contraceptive counselling for women and their partners. Honor-related behavior and violence were described as belonging to clan structures and were difficult to explain to someone representing the Swedish sexual discourse including the midwives. The participants described how honor-related practices were based on patriarchal clan structures and were mainly misogynic, affecting women’s possibilities to choose, to access, and to use contraceptives. Migrating to Sweden could, as described by one of the participants, “create a huge fight” within families with a younger generation growing up in Sweden challenging their traditional sexual values and norms, thus creating tensions between families and the Swedish society. This difference between the sexual values and norms in their home countries and in Sweden could, according to the participants, create a feeling of alienation and insecurity in the contraceptive counselling meeting with the midwife and create a feeling of mistrust towards the midwife and the state in general when living in the Swedish society. Therefore, it was confusing that Sweden as a state involved in family matters and driven by gender equality driven state did not have even more restrictive attitudes towards honor-related violence. Additionally, in order to be able to understand their wives, women in general, men and women’s bodies, and their sexual and reproductive rights some participants felt that it should be mandatory, and not by choice, for all men to visit midwives and attend information about contraceptives and sexual and reproductive health and rights.

*…” We have a collectivistic society…this is creating pressure making women not daring to act shamefully… but if the woman is acting shamefully having sex before marriage or using contraceptives… It is honor-related issues…within family…But here the state is very strong… This creates conflicts and confusion…*” …

P6

## Discussion

The study provides insight into immigrant men`s views of sexual and reproductive health and rights and contraceptives and how they perceive accompanying their wives for contraceptive counselling provided by midwives in Sweden. The overarching theme “*Balancing conflicting values and norms about sexual and reproductive health and rights including family planning*” reflected the challenges the participants perceived when having to balance two different cultures and navigating through what most often was described as contrasting values and norms regarding sexual and reproductive health and rights and use of contraception. Festinger’s [8] theory of cognitive dissonance is based on the concept that having values or attitudes contradictory to own behavior gives rise to mental conflict and an uncomfortable state of mind. According to the theory, people do not want to remain in this unpleasant state of mind and feel compelled to reduce the feeling of internal dissonance, either by justifying the behavior or by changing attitudes or actions [34]. The findings show how participants tried to ease their feelings of cognitive dissonance arising from contradictory notions about contraceptives and contraceptive counselling by suggesting how contraceptive counselling and the midwives’ work could be adjusted accordingly.

Gender equality and aspects of gender issues were mentioned throughout the interviews by the participants. Therefore, the forthcoming discussion will be viewed through a gender lens. It was not clear how gender equality was defined by the participants in the present study but their narratives revealed contradictory feelings and attitudes towards Swedish norm-giving values and laws regarding SRHR and gender equality. Previous research regarding norms and values among muslims in Sweden found that the definition of gender equality was not always a matter of men and women being equal [35, 36]. Gender equality was defined rather as men and women having the same worth which was not the same as men and women having equal access to sexual and reproductive health and rights [35, 36]. In this study, the participants questioned why a gender equal country as Sweden did not have stricter legislation promoting women’s sexual and reproductive rights. Yet, participants did not perceive it as “gender inequality” when they acted as the decision makers regarding the woman’s use of contraception, even though fertility and contraceptive use was foremost perceived as a woman’s concern. The man’s role was perceived as being the sole breadwinner, and the wife’s role was described as being a married woman with children. At the same time women’s role as reproductive “machines” having many children was described as being reinforced by the Swedish state through generous welfare systems.

Sweden is one of the most gender equal countries in the world [11]. The official Swedish definition of gender equality [37] is in line with the definition of the Sustainable Goal nr 5 [38] and is based on all people’s equal rights, conditions, opportunities, and power to shape their own lives, which also affects health positively [11]. Gender equality and women’s SRHR advocacy is a vital part of the Swedish midwife profession and the achievement of the Sustainable Development Goals [11]. However, in this study the participants perceived midwives as being protective towards women and their rights and felt sometimes unwelcome when accompanying their wives for contraceptive counselling.

Grandahl et al. [39] showed how husbands deciding about contraceptive practices and disregarding the wives’ wishes created dilemmas for the midwives (36). Arousell et al. [40] depicted how Swedish midwives were positioned in the midst of an ideological tension; the tension between gender equality ideals that should be given priority in the provision of reproductive healthcare and the fact that person centered care should be provided [40]. When providing person centered care, all individuals’ values, including beliefs in patriarchal structures and cultural and religious beliefs, should be respected [40]. Therefore, to be able to provide culturally sensitive care and to avoid conflicts in clinical encounters in a diversified society, one way could be to practice self-reflexivity regarding their own values and norms [41]. This is an interesting aspect since Swedish healthcare providers working in women’s health generally hold more liberal values regarding sexual and reproductive rights, gender equality, migration, and religion than the Swedish population at large [41].

In our study, it was evident that the participants were aware of their own values and norms and how these were affecting their perceptions of contraceptive counselling, the midwives’ role as a counsellor, and the Swedish society in general. The distance between more conservative and liberal values and norms reflects a "gap" between receiver and provider and the question is whether it is enough to be "self-reflexive" to be able to approach each other at the contraceptive counselling visit. According to our study results, the relation to the midwife was important and the feeling of trust was fundamental for how the contraceptive counselling visit was perceived. This finding is supported by previous research concerning immigrant women in Sweden who described the importance of a trustful relation to the midwife for optimal contraceptive counselling [13].

Contraceptive counselling in Sweden has mainly been provided in a one-on-one encounter since it was introduced in the mid 1970-s [42]. In this regard, it is interesting to note the participants’ view that contraceptive counselling was a matter between husband and wife, about being married and planning their family life together and never about individual needs. Critics say that by using the global term “family planning”, women’s empowerment and reproductive autonomy are diminished and that the way we are using language not only is a matter of semantics but is also creating a discourse [43, 44]. However, the Swedish midwife’s role is to provide individual contraceptive counselling and person-centered care to women and to protect her reproductive autonomy [12]. In order to comply with legal aspects, the information provided by the midwives is regulated by the Information and Secrecy Act [45]. The preservation of “secrecy” as stipulated by this act thus enables women’s contraceptive autonomy and a woman’s right to make her own reproductive choices [45]. Furthermore, midwives are subject to the Patient Safety Act [46] which stipulates that confidentiality should be maintained for each patient disregarding the patient’s relation to the accompanying person. Lack of shared awareness about the definition and purpose of the role of the midwife in contraceptive counselling could explain why the participants felt that they were not welcome and that the midwives were primarily focusing on the wife and defending her rights.

The participants stated that they wanted to be a part of contraceptive counselling. By accompanying their partner for contraceptive counselling, they gained knowledge, which in turn increased their understanding about contraceptive methods and side effects. In addition, it also opened up for conversations about a topic that was usually considered as taboo even between husband and wife. The current results also confirm findings from a recent study regarding Somali men living in Sweden, showing that men wanted to be involved in family planning (49), which may increase women’s use of contraception. Couples counselling has previously been described as opening up “doors” between husband and wife, increasing contraceptive use and decreasing unintended pregnancies [47]. As shown in previous research, midwives acknowledged the importance of male involvement, but were unclear about whose responsibility it was to involve and inform them (43). Male engagement is globally an established factor in family planning programs for improving gender equality and women’s access to contraceptives, especially in low and middle income countries [48, 49]. However, involving men in women’s reproductive autonomy, in a gender equality promoting country such as Sweden may be seen as a paradoxical. The question remains how Swedish midwifery, as a trusted profession, can improve sexual and reproductive health for both women and men (including use of contraceptives), without abandoning women’s reproductive autonomy.

Previous studies in Sweden have shown that immigrant men and women have lower levels of health literacy including sexual health and perceive the Swedish health system as difficult to navigate through [50–53]. Factors such as cultural barriers, language barriers, and lower knowledge levels about SRHR and the Swedish system are common challenges regardless of country of origin [52, 54]. This is in accordance with our study results where the participants described not knowing where to find contraceptive counselling and not knowing who the provider is. Immigrant women had similar difficulties, as shown in previous studies exploring Swedish midwives’ role as main promoters of women’s SRHR [13, 21]. Therefore, there is a need for highlighting the role of the midwife both for women and men migrating from other parts of the world who may not be used to the Swedish “model” [11, 53].

The Swedish language was described as one of the most important enabling factors for being able to receive contraceptive counselling and to increase sexual health literacy. Mistrust towards interpreters’ confidentiality and experiences of incorrect interpretations are reasons previously provided by immigrant women for rejecting the use of interpreters during contraceptive counselling [13], and the lack of well-educated interpreters with knowledge about sexual and reproductive health in Sweden is a well-known fact [50, 55]. Midwives providing antenatal care to immigrant women in Sweden described the complexity of upholding women’s autonomy when husbands who were speaking good Swedish rejected the offer of using interpreter [25].

Communicating about contraceptives and SRHR was perceived by the participants as requiring sensitivity, and it was very important to them that information be given in a sensitive manner—especially for younger generations. Today about 100 000 young students in the 9th grade are living under the threat of honor-related violence and oppression in Sweden [56]. A recent report from the Swedish Association for Sexual and reproductive health and rights Education (RFSU) [56] stated that appropriate ways of communicating about contraceptives and sexual and reproductive health and rights are indeed important in order not to alienate the listeners and make them feel stigmatized [56]. Nonetheless, the report emphasized that SRHR issues, and especially honor-related subjects must never be compromised or circumvented by teachers, healthcare providers, and others working with SRHR [56].

This study complements existing knowledge about SRHR among the immigrant population in Sweden. The study results indicate that some immigrant men find it difficult to balance cultural norms, that they do not feel welcome at the contraceptive counselling visit, yet they play an important role in their partners’ choices and their SRHR. Furthermore, the study raises important questions about men’s SRHR and the midwife’s role at a policy level. There seems to be a need to evaluate whether men should be included in midwives’ area of responsibility and if so, whether it is possible to do so without abandoning women’s SRHR. The current findings also suggest several ways to additionally provide contraceptive counselling which may improve sexual and reproductive health and rights among both immigrant men and women. For example, a more flexible approach to contraceptive counselling with outreach work may increase knowledge about the midwives’ role and enhance accessibility to SRHR services. Joint visits could be available by choice. Given that some immigrant men may affect their partners’ abilities to use contraceptives, further research about men and SRHR is therefore needed in order to improve men’s and foremost women’s sexual and reproductive health and rights. More SRHR education for men is needed and men need to be included at a policy level in SRHR strategies in order to improve women’s SRHR. Midwives need an understanding about how different values and norms are affecting people’s SRHR and midwives need to be self-reflexive to understand their own values.

### Methodological considerations

The credibility of the study was strengthened by the purposive sampling method which yielded a heterogeneous group of participants with backgrounds reflecting the non-European immigrant population in Malmö. In-depth interviews are suitable for exploring sensitive topics and perspectives [30]. However, asymmetrical power dynamics often occur between the interviewer and the participant [57]. The interviewer in the present study was a woman and participants were informed that she was a midwife by profession. These aspects may have created a willingness to share perceptions and experiences, as they all had experiences of meeting female midwives previously. However, the same information could have created a sense of shyness and reluctancy towards talking about sensitive subjects with an unfamiliar midwife and woman.

The recruitment at two maternal health clinics and a school yielded a sample that well reflected the background population. However, fewer men were recruited at the regional maternal health clinic that was located in a regular healthcare setting. According to the staff, due to the aftermath of the previous Covid-19 pandemic restrictions, men were not as present as previously. Two additional potential participants who met the inclucsion criteria were approached by the gatekeepers, but declined to participate for personal reasons. The other maternal health clinic was located in an easily accessible area and was designed as a walk-in center, which gave easy access to midwives. According to the staff from that clinic, men were accompanying their partners as usual, which could explain the higher rate of participation from that clinic in the present study. Fewer men were recruited through the regional civic and health communicators, who in turn approached both other regional and civic health communicators and immigrant students at the public school. According to the gatekeepers altogether five men declined to participate. The reasons given for non-participation were that they had not yet visited a midwife for contraceptive counselling in Sweden or that they were not married and therefore had no experience of accompanying a partner for contraceptive counselling. In total four civic health communicators participated in the study. Their unique professional knowledge about migration and civic and health communication may have contributed to the richness of the data. Furthermore, throughout the analytical process no data was left out or difficult to sort, thereby strengthening the credibility of the findings. The interview data presented a shared view of contraceptive counselling which reflected the socio-cultural views common in the Middle East and Afghanistan [19].

The first author’s prescence at the different study setting locations during the recruitment process created opportuinites for interviews to be conducted while there was a interest to participate. This was a strength because it enabled the recruitment process in a group that can be more difficult to reach for research. In order not to influence any participants choice whether to participate in the study, the first author was located in a secluded room and not visible to the partipants.

Both maternal healthcare clinics had Arabic speaking staff acting as interpreters. Three interviews were conducted with the help of the on-site interpreters and one interview with an interpreter by telephone. It is not clear if the use of interpreters (on-site interpreter or by telephone) was inhibiting the participants from speaking from their hearts. However, it is also possible that the use of an interpreter could have enabled the participants to more freely describe their inner thoughts.

All authors have previous experience in the field of sexual and reproductive health, enhancing the credibility of the study. A limitation however could be a lack of insider perspective, since the authors do not share the same background as the study participants.

Transferability of the findings to similar settings in Sweden is deemed to be good; however, it is not known to what extent the findings might be transferable to midwife counselling in other countries or to immigrant men with other backgrounds and origins.

## Conclusion

The study results indicate that men are affecting women’s abilities to use contraceptives and their right to choose if and when. Therefore, on a policy level, men’s knowledge about SRHR needs to be improved as a strategy to reach women. More research about men’s impact on women’s contraceptive use would increase knowledge and understanding about the roles of partnership and women’s and men’s sexual and reproductive autonomy. In order to reach both immigrant men and women, information about midwives and where to find contraceptive counselling is needed, as well as additional and new ways of contraceptive counselling and midwifery services.
